# Mitochondrial Respiration Correlates with Prognostic Markers in Chronic Lymphocytic Leukemia and Is Normalized by Ibrutinib Treatment

**DOI:** 10.3390/cancers12030650

**Published:** 2020-03-11

**Authors:** Subir Roy Chowdhury, Eric D. J. Bouchard, Ryan Saleh, Zoann Nugent, Cheryl Peltier, Edgard Mejia, Sen Hou, Carly McFall, Mandy Squires, Donna Hewitt, Linda Davidson, Garry X. Shen, James B. Johnston, Christine Doucette, Grant M. Hatch, Paul Fernyhough, Aaron Marshall, Spencer B. Gibson, David E. Dawe, Versha Banerji

**Affiliations:** 1 Research Institute in Oncology and Hematology, CancerCare Manitoba, Winnipeg, MB R3V 0V9, Canada; skr_chowdhury@yahoo.ca (S.R.C.); eric_bouchard@sfu.ca (E.D.J.B.); salehr@myumanitoba.ca (R.S.); znugent@cancercare.mb.ca (Z.N.); cheryl.peltier@umanitoba.ca (C.P.); mcfallc@myumanitoba.ca (C.M.); msquires@cancercare.mb.ca (M.S.); dhewitt2@cancercare.mb.ca (D.H.); lmdavidson@cancercare.mb.ca (L.D.); jjohnsto@cancercare.mb.ca (J.B.J.); spencer.gibson@umanitoba.ca (S.B.G.); ddawe@cancercare.mb.ca (D.E.D.); 2 Departments of Immunology, Max Rady College of Medicine, Rady Faculty of Health Sciences, University of Manitoba, Winnipeg, MB R3E 0T5, Canada; mejiae@myumanitoba.ca (E.M.); sen.hou@umanitoba.ca (S.H.); aaron.marshall@umanitoba.ca (A.M.); 3 Departments of Internal Medicine, Max Rady College of Medicine, Rady Faculty of Health Sciences, University of Manitoba, Winnipeg, MB R3E 3P4, Canada; gshen@ms.umanitoba.ca; 4 Children’s Hospital Research Institute of Manitoba, Winnipeg, MB R3E 3P4, Canada; cdoucette@chrim.ca (C.D.); ghatch@chrim.ca (G.M.H.); 5 Department of Medical Oncology and Hematology, CancerCare Manitoba, Winnipeg, MB R3E 0V9, Canada; 6 Departments of Physiology & Pathophysiology, Max Rady College of Medicine, Rady Faculty of Health Sciences, University of Manitoba, Winnipeg, MB R3E 3P4, Canada; 7 St. Boniface Hospital Albrechtsen Research Centre, Winnipeg, MB R2H 2A6, Canada; 8 Departments of Pharmacology and Therapeutics, Max Rady College of Medicine, Rady Faculty of Health Sciences, University of Manitoba, Winnipeg, MB R3E 3P4, Canada; pfernyhough@sbrc.ca; 9 Departments of Biochemistry and Medical Genetics, Max Rady College of Medicine, Rady Faculty of Health Sciences, University of Manitoba, Winnipeg, MB R3E 3N4, Canada

**Keywords:** chronic lymphocytic leukemia, mitochondria, oxygen consumption rates, Oroboros oxygraph, ZAP-70, B cell receptor, ibrutinib, CCL3/CCL4

## Abstract

Mitochondrial bioenergetics profiling, a measure of oxygen consumption rates, correlates with prognostic markers and can be used to assess response to therapy in chronic lymphocytic leukemia (CLL) cells. In this study, we measured mitochondrial respiration rates in primary CLL cells using respirometry to evaluate mitochondrial function. We found significant increases in mitochondrial respiration rates in CLL versus control B lymphocytes. We also observed amongst CLL patients that advanced age, female sex, zeta-chain-associated protein of 70 kD (ZAP-70^+^), cluster of differentiation 38 (CD38^+^), and elevated β2-microglobulin (β2-M) predicted increased maximal respiration rates. ZAP-70^+^ CLL cells exhibited significantly higher bioenergetics than B lymphocytes or ZAP-70^−^ CLL cells and were more sensitive to the uncoupler, carbonyl cyanide-p-trifluoro-methoxyphenylhydrazone (FCCP). Univariable and multivariable linear regression analysis demonstrated that ZAP-70^+^ predicted increased maximal respiration. ZAP-70^+^ is a surrogate for B cell receptor (BCR) activation and can be targeted by ibrutinib, which is a clinically approved Bruton’s tyrosine kinase (BTK) inhibitor. Therefore, we evaluated the oxygen consumption rates (OCR) of CLL cells and plasma chemokine (C-C motif) ligands 3 and 4 (CCL3/CCL4) levels from ibrutinib-treated patients and demonstrated decreased OCR similar to control B lymphocytes, suggesting that ibrutinib treatment resets the mitochondrial bioenergetics, while diminished CCL3/CCL4 levels indicate the down regulation of the BCR signaling pathway in CLL. Our data support evaluation of mitochondrial respiration as a preclinical tool for the response assessment of CLL cells.

## 1. Introduction

Chronic lymphocytic leukemia (CLL) is the most common leukemia among adults in Western countries [1]. It is an incurable cancer and characterized by the abnormal accumulation of mature CD19/CD5-positive monoclonal B lymphocytes [2]. Treatments may induce prolonged remissions; however, patients eventually relapse [3]. Adverse prognostic markers, such as age, male sex, cluster of differentiation 38 (CD38^+^), and zeta-chain-associated protein of 70 kD (ZAP-70^+^), predict patients who may progress rapidly and require treatment sooner [4,5]. In CLL, ZAP-70^+^ is also correlated with unmutated immunoglobulin variable heavy chain (IgVH), which is an adverse prognostic marker, and reflects increased B-cell receptor (BCR) survival signaling [6,7]. BCR stimulation increases the T-cell chemokines and chemokine (C-C motif) ligands 3 and 4 (CCL3/CCL4) in CLL cells co-cultured with nurse-like cells [8]. CCL3 secretion correlates with the expression of ZAP-70 and responsiveness of the CLL clone to BCR stimulation [9]. Furthermore, CCL3 and CCL4 have been characterized as biomarkers for BCR pathway activation in diffuse large B cell lymphoma and are reduced with BCR-directed treatment [10,11].

Altered cellular metabolism is considered a hallmark of cancer and is fast becoming a target for therapeutic intervention [12]. Mitochondria are pivotal to cell death, cell differentiation, innate immunity, hypoxia sensing, calcium metabolism, amino acids, and iron sulfur clusters, as well as heme biosynthesis [13]. Mitochondrial bioenergetics enables an objective measurement of the mitochondrial function, which may be altered in cancer and thus exploited for therapeutic benefit. To meet the needs of rapid proliferation, cancer cells change their substrate preference, including increased glucose, glutamine, and/or lipid metabolism [14,15]. Therefore, the metabolic phenotypes (glycolytic and aerobic) of cancer cells vary, and assessing the parameters linked to the hallmarks of cancer (metabolic reprogramming, metabolic phenotype, and substrate preference) will provide an increased understanding of tumor cells’ metabolic needs and help design metabolic-directed therapies [16]. Two key, measurable bioenergetics parameters that link metabolic reprogramming, the metabolic phenotype, and substrate preference in cancer cells are the glycolytic function/extracellular acidification rate (ECAR) and mitochondrial respiration/oxygen consumption rate (OCR). Carew et al. demonstrated the first evidence of an increased mitochondrial biogenesis and reactive oxygen species (ROS) level in primary CLL cells compared to normal B lymphocytes [17]. This finding was further confirmed in the compelling study by Jitschin et al., who showed that CLL cells have increased mitochondrial biogenesis, oxidative phosphorylation, and ROS generation compared to normal B lymphocytes [18]. They also found that this increase in mitochondrial biogenesis led to an elevation in respiration rates, but no difference in glycolysis, as measured by lactate production. Herishanu et al. demonstrated that CLL cells have an upregulated BCR signaling signature based on gene expression and clinically, the BCR can be targeted by phosphoinositide 3-kinase (PI3K) or Bruton’s tyrosine kinase (BTK) inhibitors [19,20]. CLL patients treated with BCR targeting agents often develop lymphocytosis with increased CLL cells in the peripheral blood [21,22]. These lymphocytes have been studied to determine their impact on clinical outcomes, as well as their biological activity. The CLL cells are viable and have downregulated BCR signaling. Despite this BCR inactivation, the cells can be stimulated or combined with other agents to evaluate combination treatments via alternate pathways [22]. Recently, Vangapandu et al. demonstrated that mitochondrial bioenergetic parameters are correlated with adverse clinical parameters and are altered in CLL by the PI3K inhibitor idelalisib [23]. However, they used extracellular flux analysis, which is an expensive technique that requires the adherence of cells to a plate and this may alter the cellular integrity [22]. In addition, cohorts from centers with referral-dependent populations may over represent rare, complex, or aggressive subpopulations compared to population-based or community-based practices.

In this study, we investigate mitochondrial respiration of CLL cells from primary patient samples with high-resolution (Oroboros) respirometry in a population-based cohort, which, to our knowledge, is the largest tested to date. We also investigate how baseline bioenergetic parameters correlate with prognostic markers and patient characteristics in untreated CLL cells. Finally, we assessed the impact of ibrutinib, which is a clinically approved BCR inhibitor, on mitochondrial respiration rates and plasma CCL3/CCL4 levels from CLL patients on ibrutinib treatment.

## 2. Results

### 2.1. Mitochondrial Bioenergetics Measured Across Two Platforms Are Equivalent

**Figure 1:**
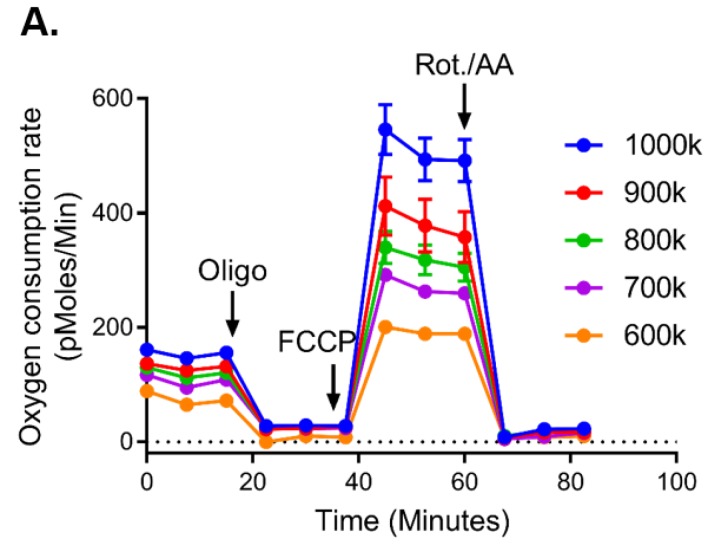

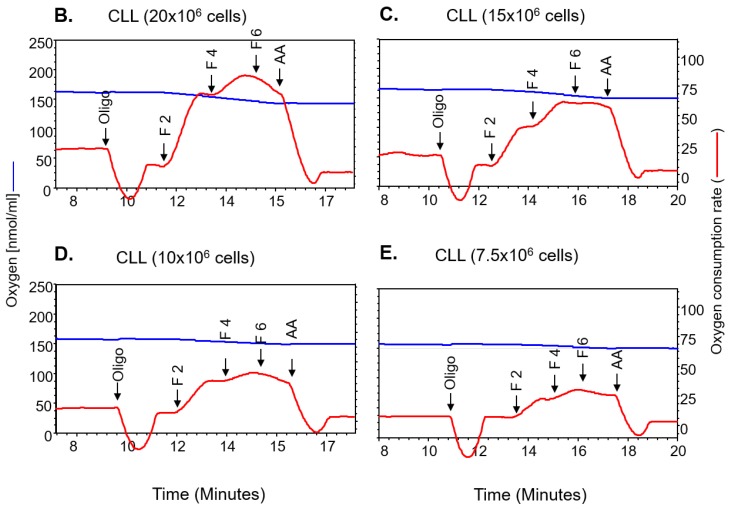
Titration of chronic lymphocytic leukemia (CLL) cell numbers by the Seahorse analyser and Oroboros oxygraph. Freshly isolated CLL cells (6 × 10^5^, 7 × 10^5^, 8 × 10^5^, 9 × 10^5^, and 10 × 10^5^) were plated to measure the oxygen consumption rate (OCR) by the XF24 Seahorse analyser. (**A**) Cells with subsequent additions of oligomycin (oligo, 1.25 µM), carbonyl cyanide-p-trifluoro-methoxyphenylhydrazone (FCCP) (2 µM), and rotenone (Rot. 1 µM)/antimycin A (AA, 1 µM). For the Oroboros oxygraph, 20 × 10^6^ (**B**), 15 × 10^6^ (**C**), 10 × 10^6^ (**D**), and 7.5 × 10^6^ (**E**) cells were added to the chamber to measure the OCR, with subsequent additions of oligomycin (oligo, 2 µM), FCCP (F2–6, 2–6 µM), and antimycin A (AA, 2 µM). Blue line indicates the level of oxygen (nmol/mL, left Y-axis) and red line indicates the oxygen consumption rate (OCR (pmol O_2_/s/indicated amount of cells, right Y-axis)).

We first demonstrate that, in order to optimize mitochondrial respiration, CLL cell titrations on both the Seahorse analyser and the Oroboros were performed. The maximal respiration rates were directly proportional to the cell number plated on the Seahorse analyser (Figure 1A: 6–10 × 10^5^ cells) and the number of cells in the Oroboros chamber (Figure 1B: 20 × 10^6^; C: 15 × 10^6^; D: 10 × 10^6^; E: 7.5 × 10^6^ cells). Using 9 × 10^5^ cells, we demonstrated that CLL cells have higher respiration rates compared to B lymphocytes on the Seahorse analyser (Figure S1A) (A: basal respiration; B: maximal respiration; C: spare respiratory capacity), and similar results were noted when using the Oroboros oxygraph while controlling for the number of cells (Figure S1A) (D: basal respiration; E: maximal respiration; F: spare respiratory capacity). Once optimized, we used the Oroboros platform and 10 million cells to perform all subsequent experiments. We also evaluated the bioenergetics parameters from isolated CLL B cells versus unselected peripheral blood mononuclear cells (PBMC) from CLL patients and found no differences in the bioenergetics profiles between the types of samples (Figure S1B). Repeat assessments were completed on 17 paired samples to ensure reproducibility across time for the same primary sample, with no differences in results when evaluated by a paired *t*-test and two-sample Kolmogorov–Smirnov test (Table S1). 

### 2.2. Adverse Prognostic Markers Predict Increased Maximal Respiration Rates

**Figure 2:**
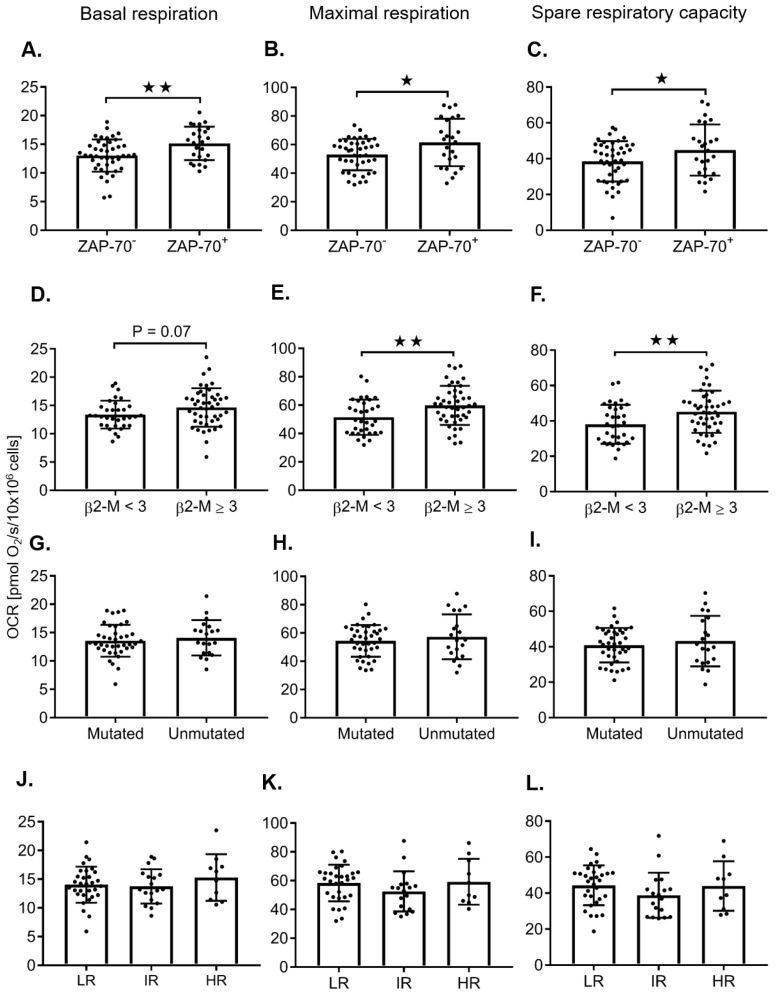
The influence of clinical prognostic markers zeta-chain-associated protein of 70 kD (ZAP-70), β2-microglobulin (β2-M), immunoglobulin variable heavy chain (IgVH) mutation status, and fluorescence in situ hybridization (FISH) on mitochondrial bioenergetic profiles in freshly isolated CLL cells. The correlation of clinical prognostic markers, ZAP-70 (ZAP-70^−^ vs. ZAP-70^+^; **A**–**C**; *N* = 41/25), β2-M (< 3 vs. ≥ 3 µM; **D**–**F**; *N* = 33/45), IgVH (mutated vs. unmutated; **G**–**I**; *N* = 39/20), and FISH (low risk, LR vs. intermediate risk, IR vs. high risk, HR; **J**–**L**; *N* = 31/18/10) with basal respiration, maximal respiration, and the spare respiratory capacity, respectively, are summarized in freshly isolated CLL cells (10 × 10^6^ cells). Values are the mean ± S.D., * *p* < 0.05, ** *p* < 0.01 (unpaired two-tailed Student’s *t-*test).

**Figure 3:**
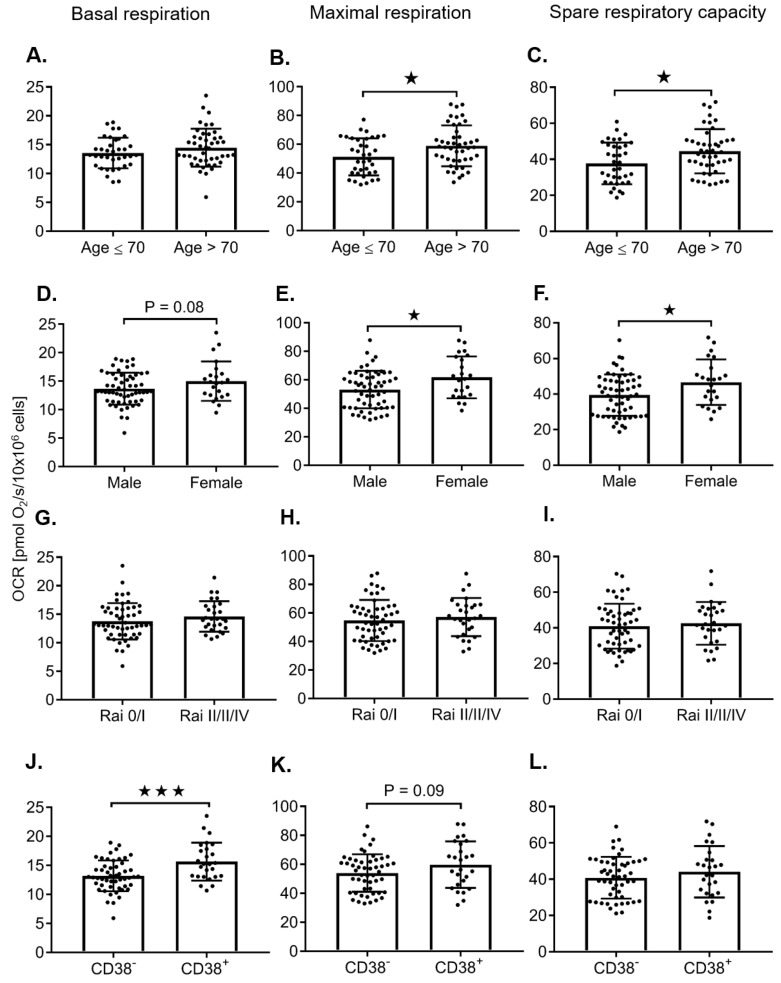
The influence of the clinical prognostic markers age, gender, Rai Stage, and cluster of differentiation 38 (CD38), on mitochondrial bioenergetic profiles in freshly isolated CLL cells. The correlation of clinical prognostic markers, age (≤ 70 vs. > 70; **A**–**C**; *N* = 36/45), gender (male vs. female; **D**–**F**; *N* = 58/23), Rai stage (0 and I vs. II, III, and IV; **G**–**I**; *N* = 53/28), and CD38 (CD 38^−^ vs. CD 38^+^ **J**–**L**; *N* = 51/26), with basal respiration, maximal respiration, and the spare respiratory capacity, respectively, are summarized in freshly isolated CLL cells (10 × 10^6^ cells). Values are the mean ± S.D., * *p* < 0.05, *** *p* < 0.0005 (unpaired two-tailed Student’s *t-*test).

The mitochondrial respiration parameters, including the basal respiration, maximal respiration, spare respiratory capacity, and respiratory control ratio, were evaluated in 81 primary CLL samples taken from individual, untreated patients while controlling for cell number (Figure 2, Figure 3 and Figure S2). When comparing untreated samples using a Student’s *t*-test, ZAP-70^+^ (≥ 20%), β2-microglobulin (β2-M) ≥ 3, age > 70, female sex, and CD38^+^ (≥20%), were correlated with higher maximal respiration rates (Figure 2B,E and Figure 3B,E), respectively. Although a trend towards higher maximal respiration rates was observed in unmutated IgVH and FISH status, this was not statistically significant (Figure 2H,K). However, when FISH tests were categorized into two groups—low risk vs. high risk (13q del versus all others (IR, HR))—high-risk FISH predicted increased maximal respiration rates (Table 1, Table S2). When evaluating patients that have 11q and or 17p deletions, there was a significant increase in basal respiration rates in female patients (Table S3A). We were not able to further evaluate the impact of FISH and IgVH in the highest risk category due to the small sample size of this subgroup. For the 13q del patients, there was no difference in bioenergetics when stratified by IgVH (Table S3B). In the univariable regression analysis, a higher dichotomous age, ZAP-70 positivity, and high β2-M were associated with increased maximal respiration, while a male sex was negatively correlated with increased maximal respiration (Table 1). Both the Pearson correlation coefficient and the Kappa score demonstrate that ZAP-70^+^, CD38^+^, and Um IgVH are all correlated (Table S3C). Advanced age increased white blood cell count (WBC), ZAP-70^+^, and elevated β2-M predict higher maximal respiration using multivariable linear regression, despite normalization of the cell number (Table 2). 

### 2.3. ZAP-70^+^ CLL Cells Have Increased Maximal Respiration

**Figure 4:**
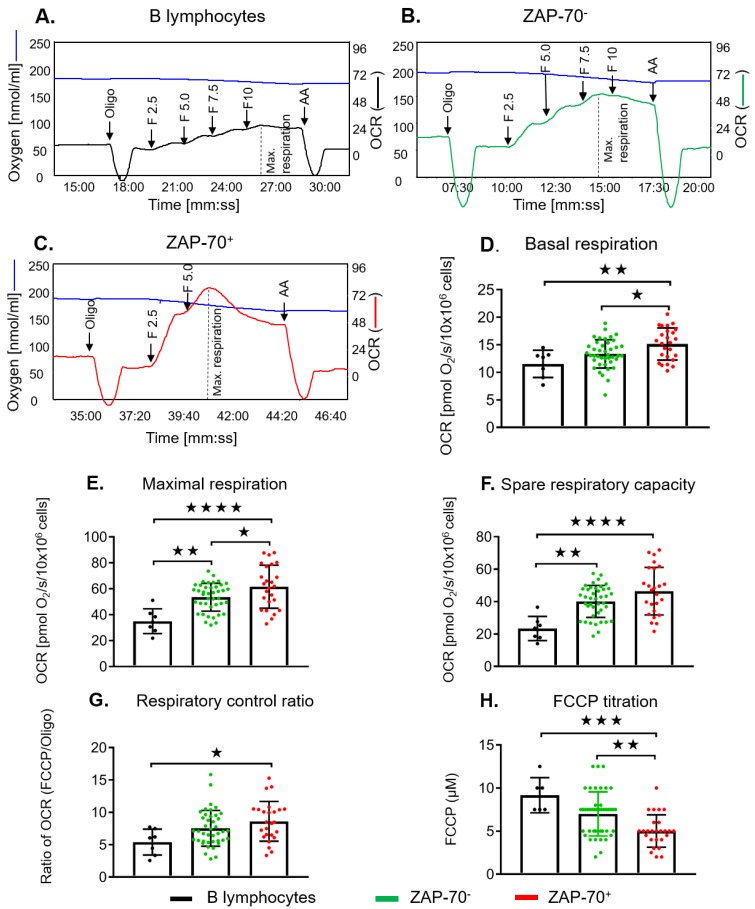
Baseline mitochondrial bioenergetic profiles in B lymphocytes and the influence of ZAP-70 on these profiles in CLL cells. The representative measurements of mitochondrial respiration rates by the Oroboros oxygraph are depicted in B lymphocytes (**A**), ZAP-70^−^ (**B**), and ZAP-70^+^ (**C**) CLL cells with subsequent additions of oligomycin (oligo, 2 µM), FCCP (F2.5–10, 2.5–10 µM), and antimycin A (AA, 2 µM). Blue line indicates the level of oxygen (nmol/mL, left Y-axis) and oxygen consumption rates, OCR (pmol O_2_/s/10 × 10^6^ cells, right Y-axis) in B-lymphocytes (black line), ZAP-70^−^ (green), and ZAP-70^+^ (red) CLL cells. Basal respiration (**D**), maximal respiration (**E**), the spare respiratory capacity (**F**), and the respiratory control ratio (**G**) are summarized in B lymphocytes (black circles, *N* = 7), ZAP-70^−^ (green circles, *N* = 41), and ZAP-70^+^ (red circles, *N* = 25), respectively. The titrations of FCCP in B lymphocytes, ZAP-70^−^, and ZAP-70^+^ CLL cells are shown (**H**). Values are the mean ± S.D., * *p* < 0.05, ** *p* < 0.01, and **** *p* < 0.0005 (one-way ANOVA with Tukey’s post hoc test).

ZAP-70^+^ has been considered a surrogate for activated BCR signaling. Based on the results from the untreated patient cohort at the time of sample collection, we went on to further characterize the effect of ZAP-70^+^ on mitochondrial respiration. ZAP-70^+^ CLL cells exhibited significantly greater basal and maximal respiration, spare respiratory capacity, and respiratory control ratio than control B lymphocytes or ZAP-70^−^ CLL cells (Figure 4A–G). When using the Oroboros oxygraph, the FCCP concentration can easily be titrated in real time for each sample, unlike the Seahorse analyser. This allows for an evaluation of the sensitivity of the electron transport chain (ETC) to FCCP, as measured by the concentration required to achieve maximal respiration (Figure 4A–C). The FCCP titration (2–12.5 µM) of ZAP-70^+^ CLL cells demonstrated that they were more sensitive to FCCP compared to ZAP-70^−^ CLL cells or B lymphocytes, achieving a maximal respiratory capacity at 5.32 ± 1.91 µM, as opposed to 8.50 ± 2.42 µM in B lymphocytes and 7.18 ± 2.43 µM FCCP in ZAP70^−^ CLL cells (Figure 4H), demonstrating their increased sensitivity to perturbations of the ETC.

### 2.4. Ibrutinib Treatment Normalizes the Mitochondrial Respiration Profile and Down Regulates the BCR Signaling in CLL Cells

**Figure 5:**
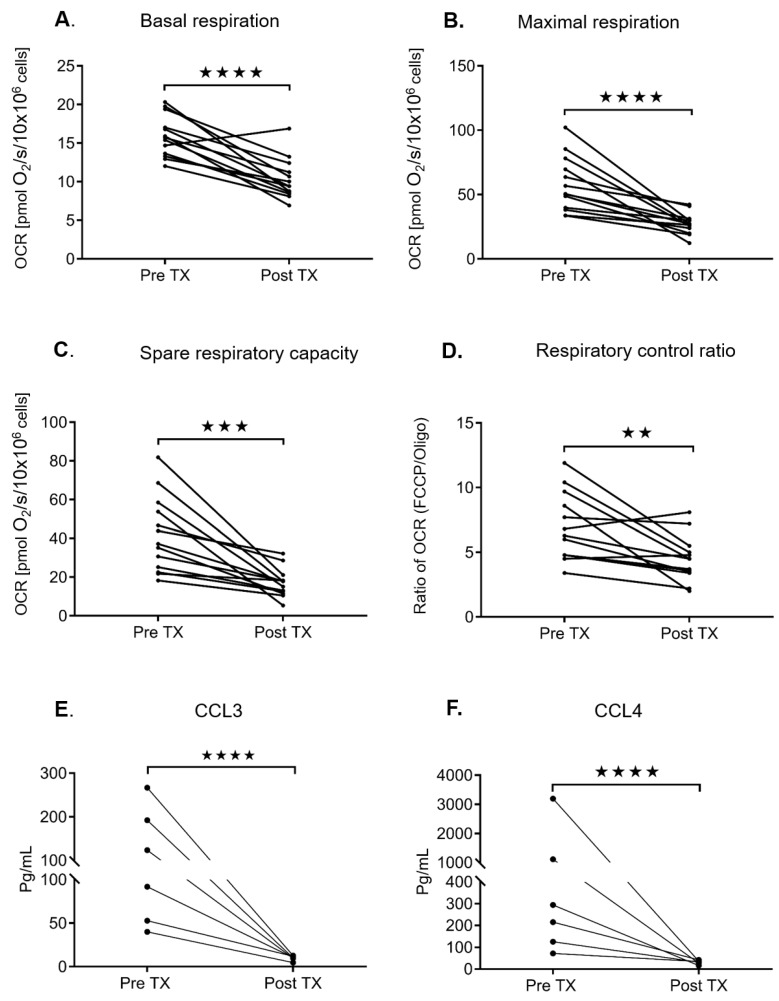
Comparison of mitochondrial bioenergetic profiles in pre- and post-ibrutinib treated CLL patients. The effect of ibrutinib treatment on basal respiration (**A**), maximal respiration (**B**), the spare respiratory capacity (**C**), the respiratory control ratio (**D**), CCL3 levels (**E**), and CCL4 levels (**F**) in pre- and post- treated ibrutinib CLL samples from the same patients is displayed. *N* = 13 for A–D and *N* = 6 for E–F. Values are the mean ± S.D., where ** *p* < 0.01, *** *p* < 0.0005, and **** *p* < 0.0001 (unpaired two-tailed Student’s *t-*test).

**Figure 6:**
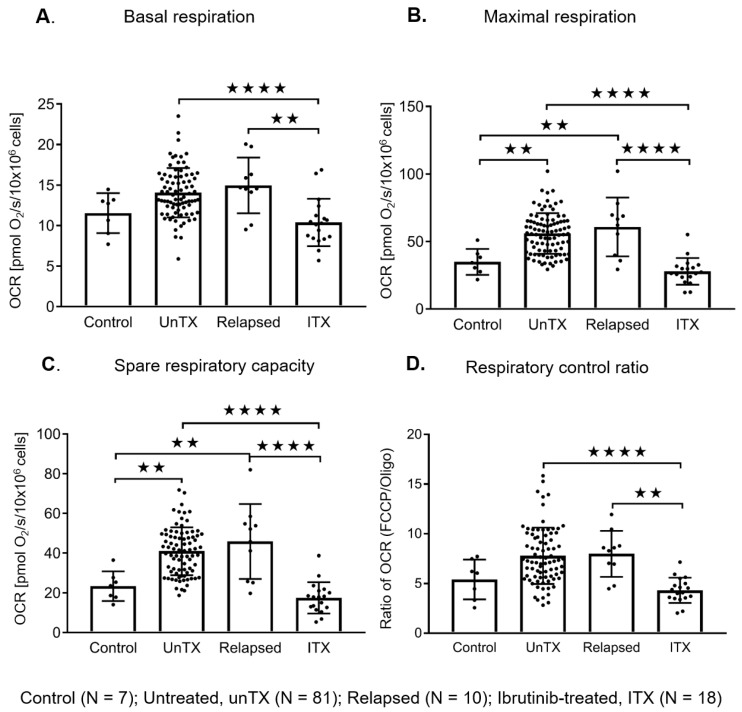
Mitochondrial bioenergetic profiles in freshly isolated B lymphocytes (Control) and CLL cells from untreated, relapsed, and ibrutinib-treated patients. Basal respiration, maximal respiration, the spare respiratory capacity, and the respiratory control ratio are summarized. Untreated, U; relapsed, R; ibrutinib-treated, I; **A**–**D**; *N* = 7/81/10/18 CLL cells (10 × 10^6^ cells), respectively. Values are the mean ± S.D., where ** *p* < 0.01 and **** *p* < 0.0001 (one-way ANOVA with Tukey’s post hoc test).

A subset of the untreated patient cohort subsequently went on to be treated with ibrutinib. When evaluating the viability of these fresh ex vivo samples, we found that untreated samples, as evaluated by annexin V and 7-AAD, were 93 % (+/− 1.297) viable, and ibrutinib-treated samples were 98% (+/− 0.232) viable (Figure S3). In samples we have paired data for, we see reductions in respiration rates from pretreatment to post treatment samples (Figure 5A–D). The plasma cytokines, and chemokine (C-C motif) ligands 3 and 4 (CCL3/CCL4) were similarly reduced in post-ibrutinib-treated samples demonstrating down regulation of the BCR pathway (Figure 5E,F). We then went on to investigate if CLL cells from untreated (U) versus relapsed patients post chemotherapy (R) or ibrutinib-treated (I) patients had similar OCR (Table 3, Table S4A–D). CLL cells obtained from patients on active ibrutinib treatment (I) showed significantly decreased bioenergetics when controlling for cell number compared to both untreated and relapsed patients. The OCR profiles of CLL cell samples from patients on ibrutinib was reduced to that of control B lymphocytes (Figure 6). All respiration parameters were significantly lower in the ibrutinib-treated group and were similar to control B lymphocytes (Figure 6, Table 3). The ibrutinib-treated patients had been receiving treatment for between 2 weeks and 12 months (Table S4D). The only differences in clinical characteristics between the untreated (U), relapsed (R), and ibrutinib-treated (I) groups, were increased β2-M in the untreated group, the ibrutinib-treated group had high-risk FISH, and both the relapsed and ibrutinib-treated groups had a higher percentage of IgVH unmutated patients (Table 3). 

## 3. Discussion

We have demonstrated that OCR is higher in CLL cells compared to control B lymphocytes, similar to previous studies [18,24]. This suggests that the mitochondrial respiration profile may serve as a biomarker for the identification of a leukemic cell. The profiles of monoclonal B-cell lymphocytosis (MBL) patients have not been evaluated systematically as cell counts are low and normalizing to cell numbers is a challenge [25]. Given that their clinical course is often similar to low-risk CLL patients, they too may have profiles different from a normal B lymphocyte; however, this remains to be determined. As technology improves, one could evaluate these parameters in a high-throughput, suspension-based assay to confirm this hypothesis. 

As others have described in smaller cohorts, we observed that alterations in OCR in primary CLL cells are associated with adverse prognostic markers, including ZAP-70 positivity, CD38 positivity, and elevated β2-M. An unexpected difference was that female sex was a strong predictor of increased maximal respiration rates. Sex differences in mitochondrial function have been documented for some tissue types, but not in CLL cells [26]. Vangapandu et al. observed differences in the extracellular acidification rate (ECAR) between sexes, but not OCR, suggesting that further investigation of this difference is warranted [23]. 

Investigations to evaluate the impact of hypoxia on CLL cells have demonstrated that there is metabolic plasticity of CLL cells with the differential production of pyruvate and the ability to protect themselves from oxidative stress [27]. Furthermore, an evaluation of the ability of CLL cells to adapt under hypoxic conditions could predict a response to future chemotherapy and thus develop a metabolic score that could inform the clinical outcome [28]. Our study did not evaluate the effect of hypoxia, but given that other pathways remain active in these ex vivo CLL cells, a future study could evaluate this with oxygen consumption rates to add to this growing literature. 

In our hands, the respiration profile was correlated with ZAP-70 expression. Vangapandu et al. have similarly demonstrated that ZAP-70^+^ CLL cells have a higher maximal respiratory and glycolytic capacity than ZAP-70^−^ CLL cells, as determined by the Seahorse XF analyser, which requires a monolayer and adhesion of cells [23]. Our ability to demonstrate the increased sensitivity to FCCP titration in ZAP70^+^ CLL cells in a suspension-based assay is novel. The Oroboros oxygraph we used enables counted cells to be measured in suspension, which allows a cell to be evaluated in an unaltered state, potentially increasing the reproducibility of future assessments [29]. Furthermore, Vangapandu et al. showed that ZAP-70^+^ and unmutated IgVH, both markers of BCR activation, were associated with higher OCRs [24]. We have demonstrated that CLL cells ex vivo from ibrutinib-treated patients remain viable and available for analysis. While we did not observe a correlation between unmutated IgVH status and OCR, we did demonstrate that targeting the BCR pathway with ibrutinib led to normalization of the mitochondrial respiration profile, suggesting that this type of test may serve as a response assessment. Since treatment with ibrutinib decreased the OCR of CLL cells to levels comparable to B lymphocytes, and that ZAP-70-positive cells are more sensitive to FCCP, one may postulate that ZAP-70^+^ cells would be more sensitive to treatments such as ibrutinib or combinations [22]. BTK is upstream in the BCR signaling pathway and its activation is often correlated with a ZAP-70-positive status [30]. A further study to evaluate this question with a larger population of ZAP-70-positive cells is warranted.

A previous study in Manitoba showed that female CLL patients have a better 5-year relative survival of 85% compared to 80% in male patients, raising the possibility that differences we see in respiration profiles by sex may translate into clinical outcome [2]. Women respond better to treatment, but often do not complete treatment and experience more side effects [31]. Women may also have a more indolent course of the disease, as they are more likely to be IgVH mutated, which is a favorable prognostic factor [31]. However, even women with unmutated IgVH do better than their male counterparts [31]. Uminski et al. have recently demonstrated that 60% of dose-reduced ibrutinib patients are female [32]. Similarly, cells from female patients may be more sensitive to therapy. One of the reasons why women may gain more benefits from less treatment is that ZAP-70^+^ cells are correlated with female sex and increased maximal respiration rates in our study. ZAP-70^+^ cells have an enhanced sensitivity to FCCP. This could explain why IgVH unmutated females do better than IgVH unmutated males given the correlation of ZAP-70^+^ with unmutated IgVH. This may suggest that females require lower drug doses to achieve an equal or greater depth of response. Sex differences and response to therapy as a function of ZAP-70^+^ in bioenergetics in CLL patients should be investigated further, but will require large numbers of patients.

One of the strengths of this study is its population-based nature; however, this resulted in a smaller proportion of high-risk molecular features in our cohort for evaluating the impact of IgVH and FISH directly. A second strength of this study is that the Oroboros oxygraph enables counted cells to be measured in suspension, which allows a cell to be evaluated in an unaltered state [29]. In the Seahorse assay, the pressure and mixing process may cause loss of cell adherence and thus loss of readouts. One of the disadvantages of the Oroboros platform is that we require a larger number of cells, but for most CLL patients, this is not an issue. While it is harder to perform Oroboros testing in normal B lymphocytes, this is often true regardless of the platform, unless one has a higher throughput Seahorse analyser. A skilled user is required for the Oroboros platform, but the lower costs of reagents make this technology much more accessible. Our work demonstrates that the Oroboros methodology can be used with primary suspension cell models as long as appropriate normalization of cell numbers occurs. Demonstrating the effectiveness of this technology enables bioenergetics to be considered a response assessment for preclinical in vitro and ex vivo evaluation with minimal cell manipulation.

## 4. Materials and Methods

### 4.1. Primary Samples

Freshly isolated peripheral blood samples from consenting CLL patients and donors without cancer were obtained (Table S4A). CLL cells from patients with a white blood cell count of < 30 × 10^9^/L were isolated for B cells using RoboSep (CD19+ kit, Stem Cell Technologies Canada Inc., Vancouver, BC, Canada). WBC counts greater than 30 × 10^9^/L were not isolated, as our internal validation demonstrated that 98% of the cells were CLL cells. All control B lymphocytes cells were isolated from non cancer controls (Supplementary Table S4B). Patient and control characteristics were provided by the Manitoba Blood and Marrow Tumor Bank and CLL CAISIS database and are listed in Table S2A (CLL) and S2B (Controls), respectively. The study was performed according to the 64th World Medical Association’s declaration of Helsinki (Fortelaza, Brasil, October 2013) and authorized by the Human Research Ethics Board at the University of Manitoba (approval number HS15746). Samples were collected from non-cancer control (*N* = 7), untreated (*N* = 81), relapsed (*N* = 10), and ibrutinib-treated (*N* = 13) subjects. Relapsed patients were patients that progressed on chemotherapy. The types of treatment prior to relapse are listed in Table S4C. The duration on treatment with ibrutinib at the time of sample measurement is listed in Table S4D. A subset of the untreated patients went on to be treated with ibrutinib. For the purposes of analysis, one patient can only be represented in one group, hence the N may vary at the time of analysis. In addition, in some cases, all molecular testing, ZAP-70, or CD38 data were not available, so only patients with available data were analyzed, and this is noted in the Ns.

### 4.2. Measurement of Mitochondrial Respiration in Isolated CLL Cells and B Lymphocytes 

**A. Respirometry:** The high-resolution Oroboros Oxygraph 2K (Oroboros Instruments, Innsbruck, Austria), a Clarke-type oxygen electrode, was used to measure mitochondrial respiration rates in CLL cells at 37 °C [33,34]. The Oroboros oxygraph has two chambers (2 mL volume) equipped with oxygen sensors. Air calibration of these oxygen sensors is performed routinely every day before starting a respirometric experiment. Freshly isolated lymphocytes were washed once in RPMI 1640 media at 335× *g* for 5 min at room temperature and re-suspended in the same media. OCR served as a surrogate for the mitochondrial electron transport chain function. OCR was measured at the baseline and following sequential treatments with the ATP synthase inhibitor oligomycin, uncoupler carbonyl cyanide-p-(trifluoromethoxy) phenylhydrazone (FCCP), to remove the pH gradient and enable maximal rates of electron transport to occur, and a combination of rotenone and antimycin A was employed to block respiratory electron flux at mitochondrial complexes I and III. After the measurement of basal respiration rates, the following chemicals were added: oligomycin (2 µM), FCCP (2–12.5 µM), and antimycin A (2 µM). Polarographic oxygen sensors monitored changes in the oxygen concentration over time. The negative time derivative was accordingly corrected for the instrumental background and displayed in real time as oxygen flux. The spare respiratory capacity was calculated by the difference between the maximal respiration achieved by uncoupler FCCP and the basal respiration. The respiratory control ratio is defined as the ratio of uncoupled respiration and oligomycin-treated respiration rates. Oroboros DatLab software was used to calculate the OCR and for the graphic presentation of experimental data. Ten million CLL cells were used for experiments, unless stated otherwise. 

**B. Extracellular Flux Analysis:** The Seahorse XF24 Analyser (Agilent Biosciences, Santa Clara, CA, USA) was also used to evaluate mitochondrial functions in B lymphocytes and CLL cells. The XF24 creates a transient 7 μL chamber in specialized 24-well microplates (Agilent Biosciences) that allows for the oxygen consumption rate to be monitored in real time [35]. Cells were pelleted by centrifugation for 10 min at 335× *g* at room temperature and re-suspended in assay media. Cells were then transferred to XF analyzer 24-well plates coated with 1.13 µg Cell-Tak (Corning, New York City, NY, USA) per well at a density of 6–10 × 10^5^ cells per well. Plates were centrifuged at 335× *g* for 10 min at room temperature, with slow acceleration and deceleration. Finally, cells were incubated at 37 °C in the non-carbon dioxide environment for 1 h prior to the assay.

To assess mitochondrial respiration, assay media consisted of unbuffered Seahorse XF DMEM (Agilent Bioscience) supplemented with 10 mM glucose and 1 mM pyruvate, at pH 7.4. OCR was measured in triplicate at the baseline and following sequential treatment with the ATP synthase inhibitor oligomycin, uncoupler FCCP, to remove the pH gradient and enable maximal rates of electron transport to occur, and a combination of rotenone and antimycin A was employed to block respiratory electron flux at complexes I and III. Oligomycin (1.25 µM), FCCP (2 µM), rotenone (1 µM), and antimycin A (1 µM) were injected sequentially through ports in the Seahorse Flux Pak cartridges. All bioenergetic parameters were calculated as mentioned above.

All chemicals and media used in the experiments for the Oroboros oxygraph and Seahorse analyser were purchased from Sigma, unless stated otherwise.

### 4.3. Cell Viability Ex Vivo in CLL Cells from Untreated and Ibrutinib-Treated Patients

Fluorescence for the detection of cell viability was assessed using a NovoCyte flow cytometer and NovoExpress software (ACEA Biosciences, San Diego, CA, USA). Cells were incubated and stained with Annexin V and 7-AAD staining, as per previously published protocols [36,37] (BD Pharmigen, San Diego, CA, USA). As a standard positive control for cell death, cells were heat shocked for 5 min at 97 °C to achieve 50% cell death (not shown). Cells were considered alive when they stain negative for both Annexin V and 7-AAD.

### 4.4. Quantification of CCL3/CCL4 Levels in Plasma from Ibrutinib-Treated CLL Patients

Plasma from pre- and post-ibrutinib-treated CLL patients was collected and then assessed for the CCL3 and CCL4 concentration using human MIP-1a and MIP-1b U-plex Assay kits (Meso Scale Diagnostics, Rockville, MD, USA) and a Mesoscale QuickPlex SQ120 (Meso Scale Discovery, Rockville, MD, USA). Assays were performed following the manufacturer’s instructions, using 4× dilutions of plasma from primary patient samples [38]. 

### 4.5. Data Analysis

Data in all figures were analyzed either by an unpaired Student’s *t*-test or one-way ANOVA with Tukey’s post hoc test using GraphPad Prism 7. A paired *t*-test and two-sample Kolmogorov–Smirnov test were used to analyse reproducibility across multiple measurements on the same individual spaced by time. Linear regression analysis was used to determine relationships between respiratory rates and prognostic markers (all tables). Variables were selected for multivariable modeling using stepwise regression, with *p* = 0.1 used to enter and 0.05 to remove a variable. Table S1 contains all the univariable linear relationships to provide background material for Table 1 and Table 2 and facilitates comparisons with other studies. Since some predictors are correlated, correction for multiple comparisons would be less reliable and may be overly conservative. Due to these concerns and the exploratory nature of our study, we chose not to adjust for multiple comparisons. The variables assessed included dichotomous age, WBC, ZAP-70, CD38, β2-M, IgVH, sex, and 3 factor FISH and Rai. These analyses were carried out with SAS 9.4 software. Differences in proportions were compared using Fisher’s exact test or a Chi-square test. Wilcoxon’s two-sample test or the Kruskal–Wallis test were used for continuous variables.

## 5. Conclusions

Overall, in this study, we have confirmed the importance of mitochondrial bioenergetic parameters of CLL cells and their association with adverse clinical prognostic markers, specifically ZAP-70 expression. We have established that high-resolution respirometry enables consistent and reproducible results with minimal technical challenges. In vivo ibrutinib treatment decreases the ex vivo bioenergetics of CLL cells to levels of normal B lymphocytes while controlling for cell number. Lower levels of CCL3/CCL4 indicate the down regulation of the BCR signaling pathway. This finding strongly supports evaluation of mitochondrial bioenergetics as a preclinical tool and potential biomarker for monitoring responses and relapses.

## Figures and Tables

**Table 1:** Univariable regression analysis of bioenergetics profiles using the dichotomous parameters. Regression analysis of significant parameters identified when evaluating prognostic markers and bioenergetic parameters in the entire cohort or cohort by sex. ZAP-70, zeta-chain-associated protein of 70 kD; CD38, cluster of differentiation 38; WBC, white blood cell count; β2-M, β2-microglobulin; PE, parameter estimate; SE, standard error. Bolded values are significant.

Dependent	Basal Respiration				Maximal Respiration				Spare Respiratory Capacity				Respiratory Control Ratio			
Variable	PE	SE	P	N	PE	SE	P	N	PE	SE	P	N	PE	SE	P	N
Age > 70 vs. ≤ 70	-	-	-		7.67	3.04	**0.014**	81	6.76	2.67	**0.013**	81	1.83	0.60	**0.0032**	81
WBC > 70 vs. ≤ 70	-	-	-		−6.54	3.19	**0.044**	81	−5.91	2.80	**0.0382**	81	-	-	-	
ZAP-70 ≥ 20 vs < 20	1.78	0.69	**0.012**	66	8.10	3.36	**0.019**	66	6.32	3.02	**0.0403**	66	-	-	-	
CD38 ≥ 20 vs. < 20	2.43	0.74	**0.0015**	71	-	-	-		-	-	-		-	-	-	
β2-M ≥ 3 vs. < 3	-	-	-		8.32	3.03	**0.0076**	**78**	7.05	2.66	0.0097	78	1.26	0.62	**0.0482**	78
Male vs. Female	-	-	-		−8.57	3.35	**0.012**	81	−7.26	2.95	**0.016**	81	-	-	-	
Age > 70 vs. ≤ 70	-	-	-		-	-	-		-	-	-		2.05	0.75	**0.0081**	58
CD38 ≥ 20 vs.< 20	2.00	0.80	**0.016**	50	-	-	-		-	-	-		-	-	-	
β2-M ≥ 3 vs. < 3	-	-	-		7.75	3.35	**0.025**	55	6.78	2.95	**0.0255**	19	-	-	-	
ZAP-70 ≥ 20 vs. < 20	2.97	1.10	**0.015**	19	16.30	6.09	**0.016**	19	13.33	5.57	**0.0286**	19	-	-	-	
CD38 ≥ 20 vs. < 20	4.05	1.50	**0.014**	21	-	-	-		-	-	-		-	-	-	
FISH 1 (0 = base)	−0.78	1.79	0.67	16	-	-	-		-	-	-		-	-	-	
FISH 2 (0 = base)	5.53	2.50	**0.046**	16	-	-	-		-	-	-		-	-	-	
FISH high risk vs. 0.1	5.81	2.35	**0.027**	16	-	-	-		-	-	-		-	-	-	

**Table 2:** Multivariable regression analysis of bioenergetics profiles using the dichotomous parameters. Regression analysis of significant parameters identified when evaluating prognostic markers and bioenergetic parameters in the entire cohort or cohort by sex. ZAP-70, zeta-chain-associated protein of 70 kD; CD38, cluster of differentiation 38; WBC, white blood cell count; β2-M, β2-microglobulin; PE, parameter estimate; SE, standard error. Bolded values are significant.

Dependent	Basal Respiration			Maximal Respiration			Spare Respiratory Capacity			Respiratory Control Ratio		
Variable	PE	SE	P	PE	SE	P	PE	SE	P	PE	SE	P
Age > 70	-	-	-	-	-	-	-	-	-	1.83	0.60	**0.003**
WBC > 70	-	-	-	−8.57	3.11	0.007	−7.60	2.73	**0.007**	-	-	-
CD38 ≥ 20%	2.43	0.74	**0.0015**	-	-	-	-	-	-	-	-	-
β2-M ≥ 3	-	-	-	10.31	3.00	0.0010	8.82	2.62	**0.001**	-	-	-
N	71	-	-	78	-	-	78	-	-	81	-	-
Age > 70	-	-	-	-	-	-	-	-	-	2.05	0.75	**0.008**
WBC > 70	-	-	-	-	-	-	-6.66	3.03	**0.032**	-	-	-
CD38 ≥ 20%	2.00	0.80	**0.016**	-	-	-	-	-	-	-	-	-
β2-M ≥ 3	-	-	-	7.75	3.35	0.025	8.51	2.95	**0.006**	-	-	-
N	50	-	-	55	-	-	55	-	-	58	-	-
ZAP-7 0 ≥ 20%	2.97	1.10	**0.015**	16.30	6.09	0.016	13.33	5.57	**0.029**	-	-	-
N	19	-	-	19	-	-	19	-	-	-	-	-

**Table 3:** Comparison of untreated, relapsed, and ibrutinib-treated CLL patients. Comparison of prognostic markers in untreated (U), relapsed (R), and ibrutinib-treated (I) CLL samples. WBC, white blood cell count; ZAP-70, zeta-chain-associated protein of 70 kD; CD38, cluster of differentiation 38; FISH, fluorescence in situ hybridization; IgVH, immunoglobulin variable heavy chain; Um, unmutated; β2-M, β2-microglobulin; PE, parameter estimate; SE, standard error. Bolded values are significant.

Variable	Untreated		Relapsed	Ibrutinib	*p*-Value		
N	78		10	13	3 Groups	Relapsed vs. Untreated	Treated vs. Untreated
Age > 70 (%)	55		40	54	0.69	0.50	0.64
WBC > 70 (%)	36		50	62	0.18	0.49	0.094
Male (%)	72		40	54	0.11	0.068	**0.045**
ZAP-70 ≥ 20 (%)	38		44	42	1	1	0.80
*n*	63		9	12	-	-	-
CD38 ≥ 20 (%)	31		38	67	0.070	1	0.065
*n*	68		8	12	-	-	-
β2-M ≥ 3.5 (%)	37		80	50	**0.031**	0.0015	**0.031**
β2-M ≥ 3.0 (%)	58		90	67	0.12	0.081	0.13
*n*	76		10	12	-	-	-
Rai II–IV (%)	33		100	62	**<0.001**	**0.0000**	0.0003
n	78		10	13	-	-	-
FISH (%)	0	53	56	15	0.6	1	0.2
	1	30	22	46	-	-	-
	2	18	22	38	-	-	-
*n*	57		9	13	-	-	-
Um IgVH (%)	33		50	73	**0.048**	0.71	**0.038**
*n*	58		9	11	-	-	-

## Supplementary Materials

The following are available online at https://www.mdpi.com/2072-6694/12/3/650/s1: Figure S1A: Mitochondrial respiration of control B lymphocytes and CLL cells by the Seahorse analyser and Oroboros oxygraph; Figure S1B: Mitochondrial respiration determined by the Oroboros oxygraph in CLL cells with low-count WBC; Figure S2: The influence of clinical prognostic markers ZAP-70, β2-M, mutational status, FISH, age, gender, Rai stage, and CD38 on the respiratory control ratio in freshly isolated CLL cells; Figure S3: Cell viability of CLL cells from untreated and ibrutinib-treated patients; Table S1: Reproducibility data; Table S2: Regression analysis of bioenergetics profiles using continuous variables; Table S3, A) Fish subgroup, B) IGVH, and C) correlation analysis; Table S4, A) CLL/SLL patient characteristics, B) control B lymphocyte donor characteristics, C) previous treatments for relapsed patients, and D) duration of ibrutinib treatment.

## Abbreviations

**AA**	Antimycin A
**ATP**	Adenosine triphosphate
**BCR**	B cell receptor
**BTK**	Burton’s tyrosine kinase
**CD38**	Cluster of differentiation 38
**CCL3**	Chemokine (C-C motif) ligand 3
**CCL4**	Chemokine (C-C motif) ligand 4
**CLL**	Chronic lymphocytic leukemia
**β2-M**	β2-microglobulin
**ECAR**	Extracellular acidification rate
**ETC**	Electron transport chain
**FCCP**	Carbonyl cyanide-p-trifluoro-methoxyphenylhydrazone
**FISH**	Fluorescence *in situ* hybridization
**IgVH**	Immunoglobulin variable heavy chain
**MBL**	Monoclonal B cell lymphocytosis
**OCR**	Oxygen consumption rates
**Oligo**	Oligomycin
**PBMC**	Peripheral blood mononuclear cells
**PI3K**	Phosphoinositide 3-kinase
**ROS**	Reactive oxygen species
**Rot.**	Rotenone
**WBC**	White blood cell count
**ZAP-70**	Zeta-chain-associated protein of 70 kD
