# Supplementary material for: Mitochondrial Respiration Correlates with Prognostic Markers in Chronic Lymphocytic Leukemia and Is Normalized by Ibrutinib Treatment
Source: Cancers (Basel). 2020 Mar 11;12(3):650. doi: 10.3390/cancers12030650 (PMC7139649; doi:10.3390/cancers12030650)
Supplement: Supplementary file 1 [file cancers-12-00650-s001.zip › Cancers-719530-Supplementary figures and table S1.pdf]

Supplementary Materials: Mitochondrial Respiration Correlates with Prognostic Markers in Chronic Lymphocytic Leukemia and Is Normalized by Ibrutinib Treatment

Subir Roy Chowdhury, Eric D. J. Bouchard, Ryan Saleh, Zoann Nugent, Cheryl Peltier, Edgard Mejia, Sen Hou, Carly McFall, Mandy Squires, Donna Hewitt, Linda Davidson, Garry X. Shen, James B. Johnston, Christine Doucette, Grant M. Hatch, Paul Fernyhough, Aaron Marshall, Spencer B. Gibson, David E. Dawe and Versha Banerji

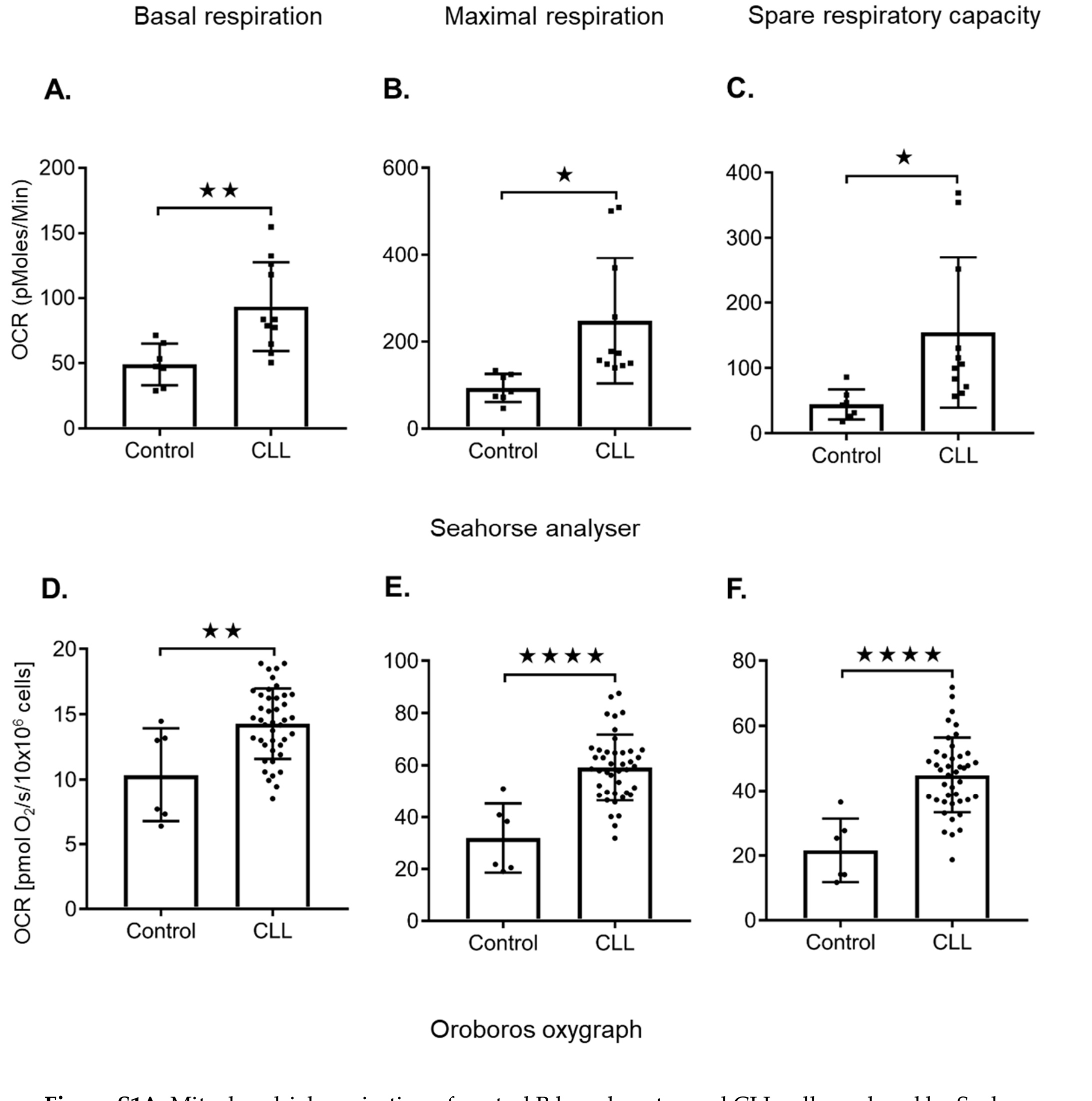

Figure S1A. Mitochondrial respiration of control B lymphocytes and CLL cells analysed by Seahorse analyser and Oroboros oxygraph. Mitochondrial bioenergetic parameters, basal respiration (**A**), maximal respiration (**B**) and spare respiratory capacity (**C**) measured by Seahorse analyser are shown in freshly isolated control B lymphocytes (N = 7) and CLL cells (N = 11) (square symbol). Basal respiration (**D**), maximal respiration (**E**) and spare respiratory capacity (**F**) measured by Oroboros oxygraph are shown in freshly isolated control B lymphocytes (N = 6) and CLL cells (N = 42) (Circular symbol). Values are mean ± S.D., * $p < 0.05$, ** $p < 0.01$, **** $p < 0.0001$ (unpaired two-tailed Student's *t*-test).

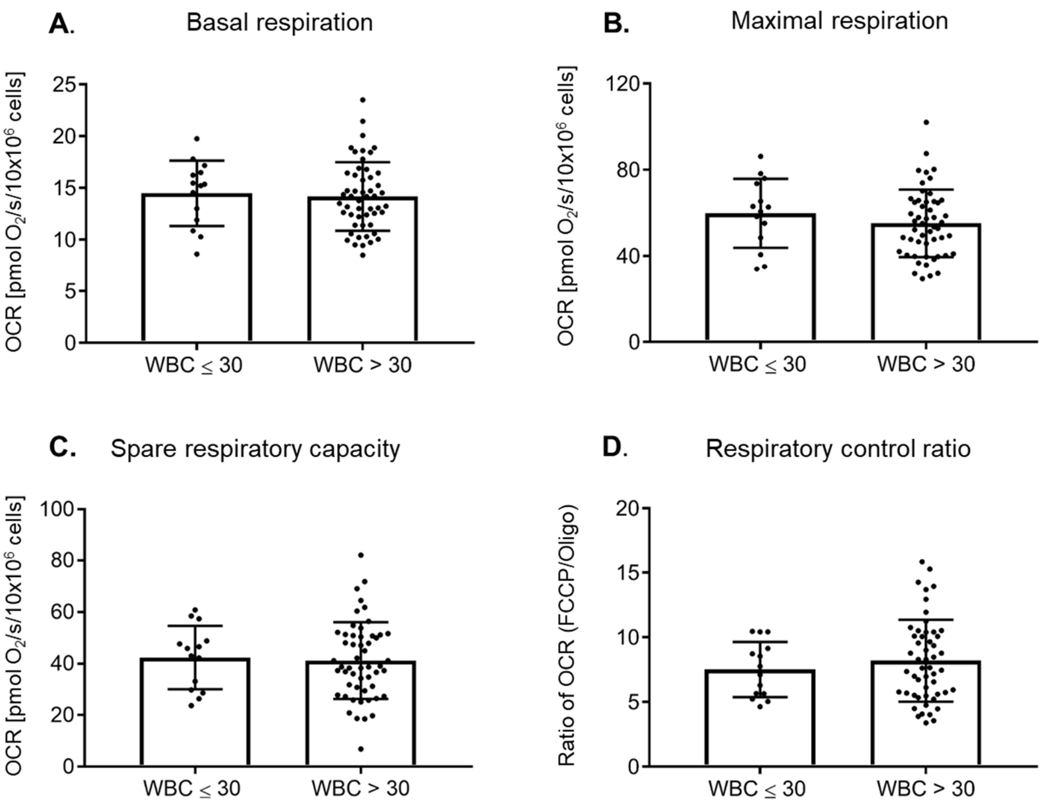

Figure S1B. Mitochondrial respiration determined by Oroboros oxygraph in CLL cells with isolated B cells or PBMC based on WBC count. Mitochondrial bioenergetic parameters, basal respiration (**A**), maximal respiration (**B**), spare respiratory capacity (**C**), and respiratory control ratio (**D**) measured by Oroboros oxygraph demonstrating no difference in respiration profiles based on isolation of CLL B lymphocytes as determined by WBC count. (WBC ≤ 30, N = 14 vs. WBC > 30, N = 52. Values are mean ± S.D., unpaired two-tailed Student's t-test.

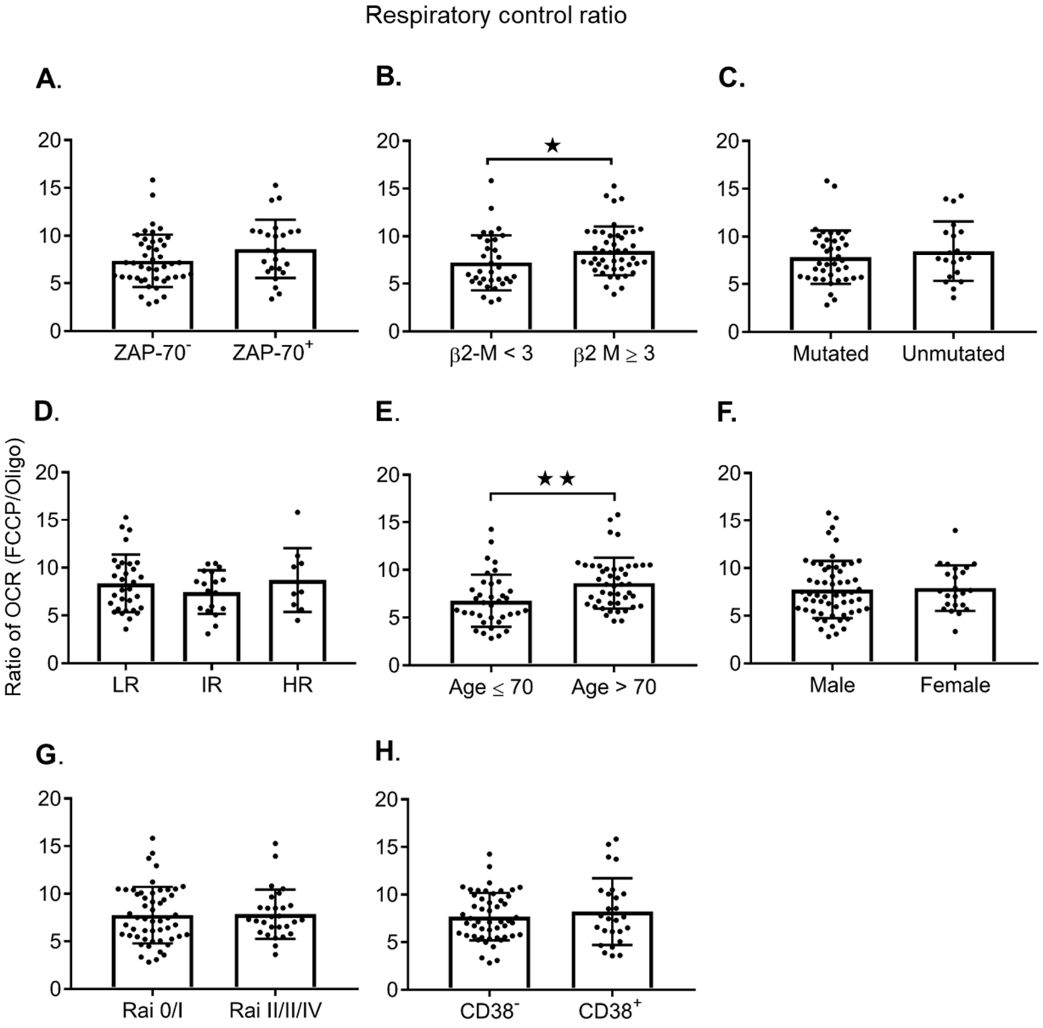

Figure S2. The influence of clinical prognostic markers ZAP-70, β2-M, mutational status, FISH, age, gender, Rai stage and CD38 on respiratory control ratio in freshly isolated CLL cells. The influence of clinical prognostic markers, ZAP-70 (ZAP-70⁻ vs. ZAP-70⁺; **A**; *N* = 41/25), β2-M (< 3 vs. ≥ 3 μM; **B**; *N* = 33/45), IgVH (mutated vs. unmutated; **C**; N = 39/20), FISH (low risk, LR; intermediate risk, IR; high risk, HR; **D**; *N* = 31/18/10), age (≤ 70 vs. > 70; **E**; N = 36/45), gender (male vs. female; **F**; *N* = 58/23), Rai stage (0 and I < vs. II, III and IV; **G**, *N* = 53/28) and CD38 (CD38⁻ vs. CD38⁺; **H**; *N* = 51/26) on respiratory control ratio are summarized in freshly isolated CLL cells (10x10⁶). Values are mean ± S.D., * $p < 0.05$, ** $p < 0.01$ (unpaired two-tailed Student's *t*-test).

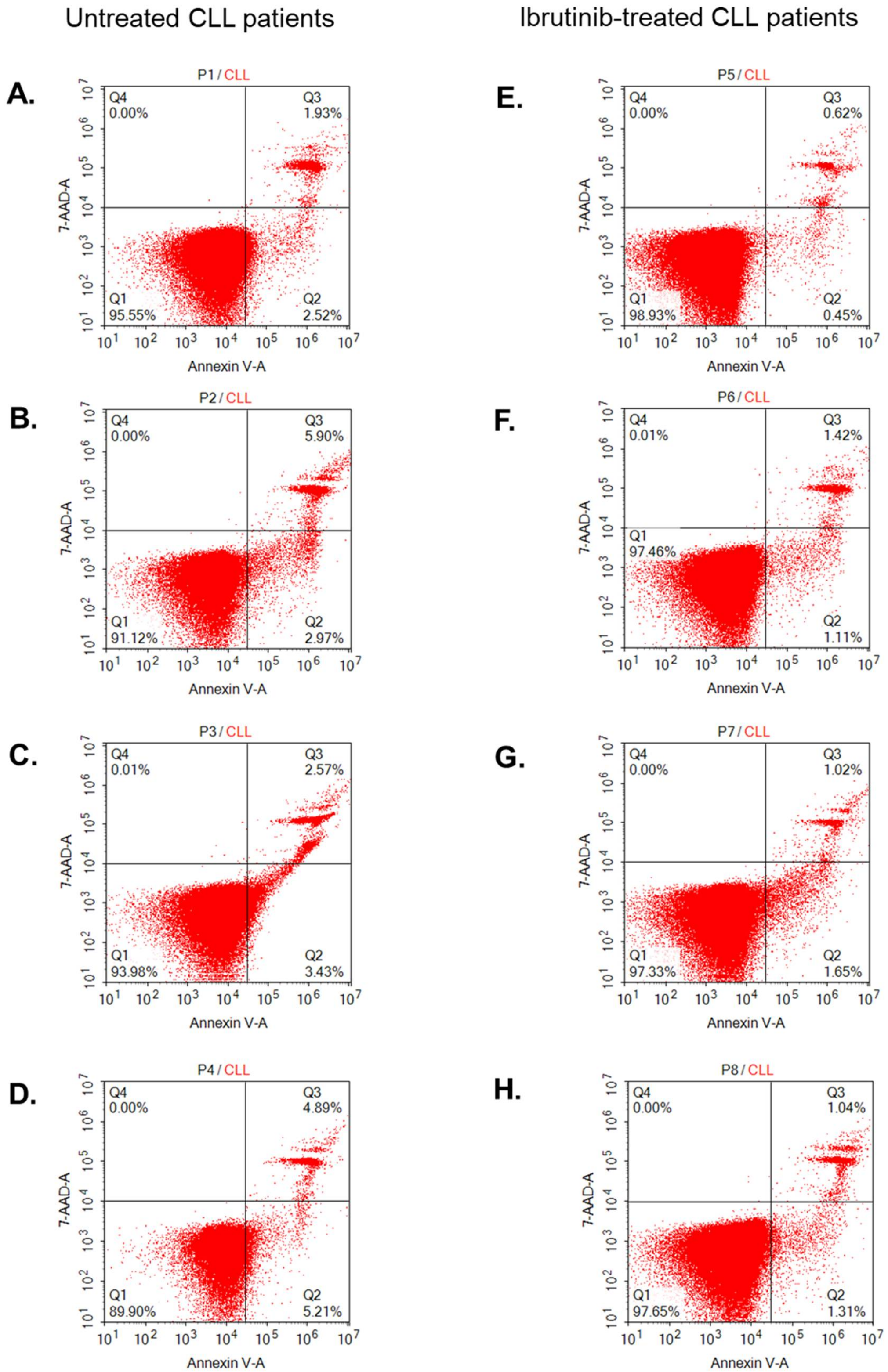

Figure S3. Ex vivo cell viability of CLL cells from untreated and ibrutinib-treated CLL patients. **Li**ve CLL cells **(%)** were assessed with Annexin V (AV) and 7-AAD in untreated CLL cells (**A**–**D**), and cells from ibrutinib treated patients (**E**–**H**) by flow cytometry. Representative panels have been divided into 4 quadrants as Q1-4. Q1: AV-, 7-AAD-; Q2: AV+, 7-AAD-; Q3: AV+, 7-AAD+; Q4: AV+, 7-AAD+.

Table S1. Reproducibility analysis of mitochondrial bioenergetics of repeated CLL samples by Oroboros oxygraph. This table demonstrates that the variation from samples from the same patients at variable time points taken repeatedly is not significant by Kolmogorov-Smirnov 2 Sample test. N, sample size; Paired T, Paired Test; Mean Diff, Mean Difference; Std Dev, standard deviation; 1st, first reading and 2nd, second reading. Bolded numbers are significant.

**Group**	**Paired T**			**Kolmogorov-Smirnov 2 Sample**	**Detectable Difference**	
	N	Mean Diff	Std Dev	*p*	*p*	
Basal respiration 1st, 2nd	17	0.0822	3.5483	0.93	0.73	± 2.568
Maximal respiration 1st, 2nd	17	0.184	6.2714	0.91	0.95	± 4.540
Spare respiratory capacity 1st, 2nd	17	0.1017	6.7036	0.95	0.9998	± 4.853
Respiratory control ratio 1st, 2nd	17	1.0373	3.026	0.18	0.24	± 2.190

Tables S2–S4 are in a separately excel

© 2020 by the authors. Licensee MDPI, Basel, Switzerland. This article is an open access article distributed under the terms and conditions of the Creative Commons Attribution (CC BY) license (http://creativecommons.org/licenses/by/4.0/).
